# Hepatitis B and C prevalence among the high risk groups of Pakistani population. A cross sectional study

**DOI:** 10.1186/0778-7367-70-9

**Published:** 2012-04-26

**Authors:** Abdul Rauf Memon, Kashif Shafique, Ashraf Memon, Agha Umer Draz, Mohammad Uzair Abdul Rauf, Salahuddin Afsar

**Affiliations:** 1 Department of Medicine, Dow University of Health Sciences and Civil Hospital Karachi, Karachi, Pakistan; 2 Institute of Health & Wellbeing, Public Health, University of Glasgow, 1 Lilybank Gardens, Glasgow, G12 8RZ, UK; 3 Sindh Aids Control Program, Karachi, Pakistan; 4 Department of Pathology, Civil Hospital Karachi, Karachi, Pakistan; 5 Dow Medical College, Dow University of Health Sciences, Karachi, Pakistan

**Keywords:** Hepatitis B, Hepatitis C, High Risk Group, Pakistan

## Abstract

**Background:**

Hepatitis B and C cause significant morbidity and mortality worldwide. Little is known about the existence of hepatitis B and C among high risk groups of the Pakistani population. The present study was conducted to determine the prevalence of Hepatitis B and C in high risk groups, their comparison and the possible mode of acquisition by obtaining the history of exposure to known risk factors.

**Methods:**

This cross sectional study was carried out in Karachi, from January 2007 to June 2008.

HBsAg and Anti HCV screening was carried out in blood samples collected from four vulnerable or at risk groups which included injecting drug users (IDUs), prisoners, security personnel and health care workers (HCWs). Demographic information was recorded and the possible mode of acquisition was assessed by detailed interview. Logistic regression analysis was conducted using the STATA software.

**Results:**

We screened 4202 subjects, of these, 681 individuals were reactive either with hepatitis B or C. One hundred and thirty three (3.17%) were hepatitis B reactive and 548 (13.0%) were diagnosed with hepatitis C. After adjusting for age, security personnel, prisoners and IV drug users were 5, 3 and 6 times more likely to be hepatitis B reactive respectively as compared to the health care workers. IDUs were 46 times more likely to be hepatitis C positive compared with health care workers.

**Conclusion:**

The prevalence of hepatitis B and C was considerably higher in IDUs, prisoners and security personnel compared to HCWs group. Hepatitis C is more prevalent than hepatitis B in all these risk groups. Prevalence of hepatitis C increased with the increase in age. Use of unsterilized syringes, used syringes, body piercing and illicit sexual relations were found to be important associated risk factors for higher prevalence of Hepatitis B and C in these groups.

## Background

Both hepatitis B and C are common infections affecting masses and are the leading causes of chronic liver disease. Hepatitis B affects 350 to 400 million people worldwide and accounts for 1 million deaths from cirrhosis, liver failure and hepatocellular carcinoma [1]. Hepatitis C affecting 130–170 million people worldwide, is a leading cause of liver related deaths and most frequent reason for liver transplantation [2,3]. About two third of people suffering from hepatitis B and C are residing in the developing countries [4].

Pakistan is in the intermediate zone of hepatitis B and C prevalence areas. The prevalence is described as around 4% and 6% respectively [5,6]. Higher prevalence of Hepatitis B and C has been reported in certain areas of Pakistan in small scale studies [7]. The reason for high prevalence is explained by several factors including transfusion of unscreened/improperly screened blood or blood products [8], use of injections by unsterilized syringes in general practice by quacks [9], nose prick or ear prick by unsterilized needles and frequent visits to barbers for shaving purposes [10,11]. Use of unsterilized syringes among intravenous drug users is a significant risk factor of Hepatitis B and C globally [12,13]. Pakistan is the country with highest number of intramuscular injections injected per person per year [14]. Horizontal transmission in children appears to contribute more than vertical transmission for prevalence of chronic hepatitis B in this region. Unprotected sex accounts for lesser number of cases than those in developed countries.

Recently, Pakistan Medical and Research council has conducted a community-based study showing a reduction in the prevalence rate of hepatitis B & C to 2.5% and 4.9% respectively, in the general population [15]. This may be due to an increase in public awareness regarding preventive strategies and inclusion of hepatitis B vaccine in National immunization program since 2000. Higher prevalence however, have been reported in certain high risk groups and in family members of hepatitis C patients [16,17]. Data of prevalence in high risk groups is sparse and a comparison of different risk groups is required to formulate national health policies and preventive strategies. Therefore the present study was conducted to determine the prevalence of Hepatitis B and C in high risk groups, their comparison and possible mode of acquisition.

## Methods

The study was conducted from January 2007 to December 2008 as part of an effort to control and prevent viral hepatitis in the province. Four vulnerable or at risk population groups were identified for this cross-sectional study. These included IV drug users, prisoners, security personnel and healthcare workers. Health care workers were included in high risk groups due to higher exposure to hepatitis patients, routinely reported needle prick injuries and low uptake of hepatitis vaccine. This group is of particular importance because, infected health care workers can transmit the infection to uninfected patients admitted to hospital, which can further spread the disease in society.

We compiled a database of prisoners in Landhi Jail, health care workers of the Civil Hospital Karachi, IDUs registered by the Referral Laboratory of Sindh AIDS Control Program and security personnel from a large private company. A unique identification number was assigned to all individuals in this database. A computerized program was run to generate a random sample of the individuals included in this database. All identified individuals were then approached for a detailed interview and blood sample collection. The response rate was 83% in this study with a total of 4202 individuals included in this analysis.

We estimated the sample size to measure a 2% difference of hepatitis prevalence (assuming 4% prevalence) [5,6] between groups at 0.05 significance level using a two-sided comparison and the power of 90%. Sample size was computed for both the *chi-squared test* using the Yates’ continuity correction and Fisher Exact test. A sample of 3252 participants was the minimum number required to be accrued in order to perform this survey.

Among HCWs, only staff from exposure-prone procedures (EPP) was included in the study. These included intensive care unit staff, surgeons, nursing staff, phlebotomists, doctors, nurses and other paramedical staff providing direct care to the patient. Demographic information of screened people was recorded along with history of risk factors. A team of laboratory workers was deputed to collect samples from these groups. All samples were taken after obtaining the informed consent. Consent was also obtained from the institutional heads with assurance of confidentiality and provision of treatment from the hepatitis control program. The study protocol was reviewed and approved by an independent ethics committee. Sample collection was carried out at different sites for the groups as indicated. Five milliliter of venous blood was collected in gel vaccutainer tubes (yellow top) using aseptic technique. Blood samples were transported to the designated lab for further processing.

HBsAg and HCV Antibody tests were performed at the central lab of the Civil Hospital Karachi (CHK) using the CMEIA (Chemoluscent micro particle enzyme immunoassay) method on blood samples from HCW, prisoners and security personnel. Samples from IDUs were tested at the Referral laboratory of Sindh AIDS Control Program by ELISA Methods using Bio-Rad Monolisa Antigen-antibody Coombo test kits.

Data were recorded and analyzed using the Stata Software Version 11 (StataCorp, College Station, TX, USA). We categorized the age using 10-year groups from 21–30, 31–40, and 41–50 years old. The means between groups were compared using the independent sample *t* test and analysis of variance (ANOVA) with bonferroni adjustments for multiple comparisons was done. We explored the association between age group, gender and risk group by using the logistic regression model. Health-care worker group was taken as a reference category when investigating the relationship between risk group and hepatitis B and C. As this group may have prevalence of hepatitis B and C approximate to the background population or may be slightly higher than that. We constructed two different models for hepatitis B and Hepatitis C. Age was found to be significant on univariate analysis and was therefore included in the final multivariate model to make appropriate adjustments. The level of significance was set at 95% (p value < 0.05).

## Results

We screened 4202 subjects for hepatitis B and C, out of these, 3637 were males and 565 were females. Female participants were only available to participate in the health care workers group. The mean age of participants was 41.07 ± 6.06. On average, females were significantly (p < 0.001) younger than males (38.6 versus 41.4 years). There was no significant difference of age between those who were hepatitis B reactive (42.1 ± 6.09 years) compared with non-reactive subjects (40.9 ± 5.97 years) (Table 1).

**Table 1:** Baseline characteristics of subjects screened for Hepatitis B and C

**Characteristics**	**Number (n)**	**%**
**Total participants (n)**	4202	_
**Age (years), mean (s.d.)**	41.07 (6.06)	_
21–30	270	6.43
31–40	1609	38.29
41–50	2323	55.28
**Gender**		
Male	3637	86.55
Female	565	13.45
**Risk group**		
Health care workers	1051	25.00
Security personnel	457	10.88
Prisoners	2287	54.43
Intra venous drug users	407	9.69

**Figure 1:**
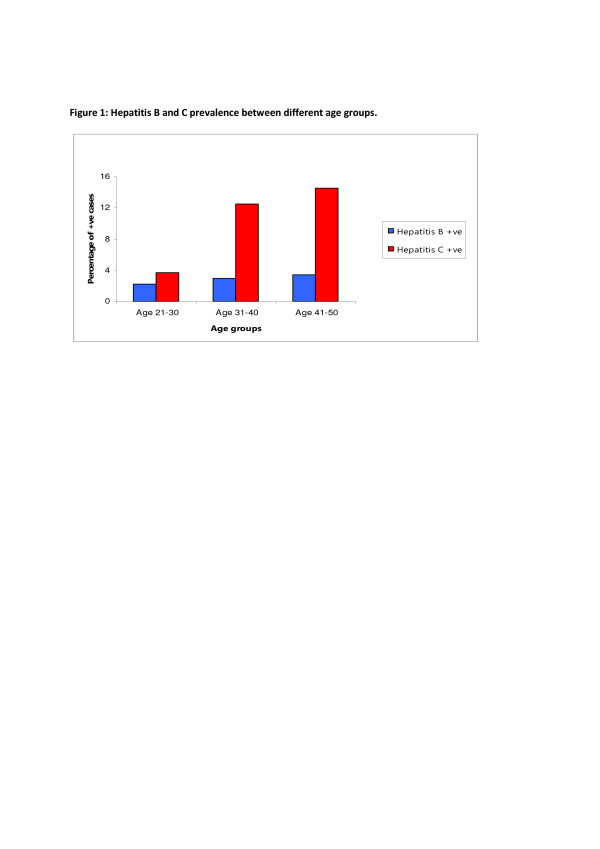
Hepatitis B and C prevalence between different age groups.

A total of 681 participants were reactive either with hepatitis B or C. One hundred and thirty three (3.17%) were hepatitis B reactive and 548 (13.0%) were diagnosed with hepatitis C. The proportion of hepatitis B reactive cases was fairly similar across different age categories; however the frequency of hepatitis C reactive cases was significantly higher among individuals of ages between 41 to 50 years compared to the individuals of age 21–30 years (p-value < 0.001) (Figure 1).

Mean age of prisoners and IDUs screened in this study was significantly higher (p < 0.001) compared with health care workers and security personnel. Difference observed in the frequency of hepatitis C across different age categories was explored after adjusting the effect of the risk group. Prevalence of hepatitis B and C differed significantly between groups. Prevalence of hepatitis B and C was lowest among HCWs (hepatitis B 1.0%, hepatitis C 2.9%), while prevalence of hepatitis B and C was highest among IDUs (hepatitis B 5.7%, hepatitis C 68.3). Prevalence of hepatitis B was 1.1% higher among security personnel compared with prisoners, while there was no significant difference in prevalence of hepatitis C between these two groups (Table 2). Individuals between the ages of 41–50 years were 69% more likely to have reactive hepatitis C as compared to those between the ages of 21–30 years after adjusting the group effect (OR 1.69, 95% CI 0.85–3.39).

**Table 2:** Association of hepatitis B and C infection with age and high risk group

			**Hepatitis B**			**Hepatitis C**	
**Characteristics**	**Total**	**Hepatitis B reactive,n (%)**	Adjusted odds ratios * (95% Confidence interval)	**p value**	**Hepatitis C reactive,n (%)**	Adjusted odds ratios * (95% Confidence interval)	**p value**
							
**Age (years)**							
21–30	270	6 (2.2)	1	_	10 (3.7)	1	_
31–40	1,609	48 (3.0)	0.95 (0.39–2.33)	0.91	201 (12.5)	1.67 (0.84–3.34)	0.13
41–50	2,323	79 (3.4)	1.03 (0.42–2.51)	0.94	337 (14.5)	1.69 (0.85–3.39)	0.12
**Risk group**							
Health care workers	1,051	10 (1.0)	1	_	30 (2.85)	1	_
Security personnel	457	21 (4.6)	5.03(2.35–10.78)	0.001	41 (8.97)	3.35 (2.06–5.45)	<0.001
Prisoners	2,287	79 (3.5)	3.69 (1.87–7.24)	0.003	199 (8.7)	3.04 (2.04–4.53)	<0.001
Intra venous drug users	407	23 (5.7)	6.16 (2.86–13.24)	<0.001	278 (68.3)	46.60 (28.25–76.84)	<0.001

* Odds ratio presented after mutual adjustments of all covariates mentioned in the table.

In multivariate analysis there were no significant differences observed in the likelihood of hepatitis B between different age categories and sex. However, after adjusting for age, security personnel, prisoners and IDUs were 5, 3 and 6 times more likely to be hepatitis B reactive respectively compared with the health care workers group (Table 2).

In multivariate model (adjusting for sex and risk group) for hepatitis C, age had no significant impact on the likelihood of being reactive. Furthermore, after adjusting for age, security personnel and prisoners had significantly higher odds of being hepatitis C positive (p = 0.003) (Table 2). Injecting drug users were 46 times more likely to be hepatitis C positive compared to health care workers after multivariate adjustments (Table 2).

Regarding risk factors, significantly high proportion of prisoners and security personnel was found to had illicit sexual relations i.e. 31% and 36% respectively, compared with HCWs and IDUs (p-value 0.01). A history of blood transfusion was also positive among a significant proportion of participants between the risk groups. Injecting drug users were significantly more likely to have a history of blood transfusion compared with other three risk groups (p value 0.03). Interestingly, the use of heroin injections was significantly higher among prisoners (39%) compared with security personnel and health care workers group (p-value 0.001), summary of other risk factors have been provided in Table 3.

**Table 3:** Distribution of risk factors for hepatitis B and C among the high risk groups

					**High risk groups**			
**Characteristics**	**Health care workers**		**Prisoners**		**Security personnel**		**IV drug users**	
	***N***	**(%)**	***N***	**(%)**	***N***	**(%)**	***N***	**(%)**
**Total participants**	1051		2287		457		407	
**Age, mean (s.d.)**								
**Marital status**	38.6	(6.8)	42.5	(5.1)	38.3	(6.8)	42.5	(4.9)
Umarried	210	(20)	1464	(64)	247	(54)	195	(48)
Married	841	(80)	823	(36)	210	(46)	212	(52)
**Literacy level**								
Iliterate	74	(07)	1418	(62)	434	(95)	175	(43)
Literate	977	(93)	869	(38)	23	(05)	232	(57)
**Current living arrangement**								
Home	1009	(96)	0	(0.0)	9	(02)	236	(58)
Streets	0	(0.0)	0	(0.0)	0	(0.0)	106	(26)
Shrine	0	(0.0)	0	(0.0)	0	(0.0)	20	(05)
Away from home	42	(04)	2287	(100)	448	(98)		
**Illicit sexual relation**								
No	1009	(96)	1578	(69)	292	(64)	330	(81)
Yes	42	(04)	709	(31)	165	(36)	77	(19)
**Injection practices**								
Blood transfusion	21	(02)	91	(04)	9	(02)	33	(08)
Body piercing	74	(07)	480	(21)	55	(12)	12	(03)
Needle stick injury	231	(22)		__		__	102	(25)
Injection practices								
* heroin injections*	0	(0.0)	892	(39)	9	(02)	269	(66)
* injection more than 3 times/day*	0	(0.0)	46	(02)	0	(0.0)	65	(16)
* injection with used syringe*	0	(0.0)	0	(0.0)	0	(0.0)	90	(22)
* shared syringe/needle on last time*	0	(0.0)	0	(0.0)	0	(0.0)	118	(29)

## Discussion

Hepatitis B and C are endemic diseases in Pakistan and carry high morbidity and mortality. This study was conducted in four at risk or vulnerable groups and the results indicate higher prevalence of both hepatitis B and C among IDUs, prisoners and security personnel compared with HCWs. Except the healthcare workers group the prevalence of hepatitis B and C is much higher in all other risk groups compared to general population. Previous studies on HCWs in this region have shown prevalence similar to that of the general population [18]. Our study however revealed a much lower prevalence in the health care workers group, which may be due to the better uptake of vaccination or less exposure to the risk factors of hepatitis B and C. Studies on HCWs from India have already shown a low prevalence of hepatitis B and C infection due to vaccination for hepatitis B and better awareness regarding preventive strategies for hepatitis C [19].

High prevalence of hepatitis C and B in IV drug users is already reported from all over the world including the Indian sub-continent [19,20]. Previous studies from Pakistan on IDUs have also shown a high prevalence of hepatitis C, B and HIV infections among IDUs [21,22]. Like previous studies our study has also shown a significantly higher prevalence of hepatitis C in this groupcompared to hepatitis B. We have also observed in our study a higher prevalence of hepatitis C among aging group (41–50) in all categories compared to younger group (21–30) and similar results are shown in studies from India [19,23]. These can be explained on the basis of multiple risk exposures of aging groups including injections with unsterilized syringes, blood transfusions and invasive procedures.

The prevalence of hepatitis C and B in this study is much higher in IDUs and prisoners as compared to HCWs. This might be explained by the increase in exposure to unsterile syringes, sharing of syringes, illicit sexual practices, body piercing and low literacy among IDUs and prisoners. Both IDUs and prisoners are overlapping groups as most prisoners are injecting drugs and also involved in illicit sexual practices [23,24]. A high prevalence of hepatitis B and C has also been observed in security personnel in our study. This section of the population had not been studied in previous local studies but in this study 98% of security personnel were living away from their homes and two third of them had illicit sexual relations. There is a fair chance that these men may have developed the disease due to illicit sexual behavior due to their release from family restraints. This group of population had significantly higher risk of contracting hepatitis B and C as compared to the general population and required to be involved in preventive programs. There is some evidence, where, army recruits have shown a higher prevalence of hepatitis Bcompared withthe prevalence in the general population [25]. A high prevalence of hepatitis B and C among security personnel may also be explained on the basis of health seeking behavior with the risk of getting injection with unsterilized or reused syringes although our data did not show evidence of injection practices in this population group. Most of the security personnel in this study belonged to rural areas. They prefer injections for minor ailments and want to get rid of symptoms immediately to rejoin their duties. As chronic hepatitis B and C are common causes of Hepatocellular Carcinoma in Southeast Asia, effective hepatitis B vaccination programs, prevention of hepatitis C by screening blood donors, universal precautions against blood contamination in healthcare settings and reducing hepatitis C transmission from IDUs are important strategies to reduce hepatocellular carcinoma incidence [26].

There are few limitations in our study which are worth mentioning. We used cross sectional design for this study, participants included were those who were asymptomatic and were survivors of the disease, so there might be some survivor bias as patients with more aggressive and end stage disease might have died earlier and might not be included. We could not study the hepatitis B vaccination status in all the high risk groups and can only presume it to be very low compared to health-care workers group and this may be an additional reason for high prevalence of hepatitis B in other groups. Also we have not studied certain risk groups which has been included by other researchers like professional blood donors [27], patients receiving frequent transfusion of blood and blood products [16] and patients on haemodialysis [17]. A major limitation of this study is the unequal sizes of risk groups; this was mainly due to the difficulty in approaching IDUs and security personnel for the screening program. Similarly, females only took part from health care group while we could not gain access to female groups in the rest of the three risk groups. Although, our study showed a significantly higher risk of hepatitis B and C among IDUs, prisoners and security personnel,some groups had smaller number of cases and confidence intervals were wide, these may be chance findings which need further confirmation. Also, future studies should attempt to include females of high risk groups and other vulnerable groups need to be examined like garbage collectors, immigrants especially from Afghanistan and internally displaced people.

Our study highlights the importance of the control of chronic hepatitis B and C in certain high risk groups as these pools provide a trigger for the spread of disease in the community. Vaccination for hepatitis B and preventive strategies for hepatitis C will not yield desirable results unless it is controlled in these vulnerable groups as a spillover of disease in the community will continue.

## Conclusion

The present study showed a higher prevalence of hepatitis C and B in IDUs and prisoners compared to HCWs group. The prevalence of Hepatitis C is considerably higher in these groups than hepatitis B. The prevalence of hepatitis B and C is also significantly higher in security personnel than HCWs group. Increase in age increases the risk of hepatitis C in vulnerable groups. Useage of unsterilized syringes, used syringes, body piercing and illicit sexual relations are the primary reasons for higher prevalence of hepatitis B and C in these groups.

## Competing interests

The authors declare that they have no competing interest.

## Authors’ contributions

ARM, KS and AM conceived the idea and design of this study. UD and MU were involved in the drafting of questionnaires and collection of data. KS, MU analyzed the data; all authors were involved in the interpretation of data. ARM written the initial draft of the manuscript and all authors were involved in critical revision of manuscript for important intellectual content. All authors approved the final draft for publication.
